# Comparative profiling of immune genes improves the prognoses of lower grade gliomas

**DOI:** 10.20892/j.issn.2095-3941.2021.0173

**Published:** 2021-10-09

**Authors:** Zhiliang Wang, Wen Cheng, Zheng Zhao, Zheng Wang, Chuanbao Zhang, Guanzhang Li, Anhua Wu, Tao Jiang

**Affiliations:** 1 Department of Neurosurgery, Beijing Neurosurgical Institute, Capital Medical University, Beijing 100050, China; 2 Department of Neurosurgery, The First Hospital of China Medical University, Shenyang 110001, China; 3 Department of Neurosurgery, Beijing Tiantan Hospital, Capital Medical University, Beijing 100050, China

**Keywords:** Lower grade glioma, immune, gene pairs, signature, prognosis

## Abstract

**Objective:**

Lower grade gliomas (LGGs), classified as World Health Organization (WHO) grade II and grade III gliomas, comprise a heterogeneous group with a median survival time ranging from 4–13 years. Accurate prediction of the survival times of LGGs remains a major challenge in clinical practice.

**Methods:**

We reviewed the expression data of 865 LGG patients from 5 transcriptomics cohorts. The comparative profile of immune genes was analyzed for signature identification and validation. In-house RNAseq and microarray data from the Chinese Glioma Genome Atlas (CGGA) dataset were used as training and internal validation cohorts, respectively. The samples from The Cancer Genome Atlas (TCGA) and GSE16011 cohorts were used as external validation cohorts, and the real-time PCR of frozen LGG tissue samples (*n* = 36) were used for clinical validation.

**Results:**

A total of 2,214 immune genes were subjected to pairwise comparison to generate 2,449,791 immune-related gene pairs (IGPs). A total of 402 IGPs were identified with prognostic values for LGGs. The *HOXA9*-related and *CRH*-related scores facilitated identification of patients with different prognoses. An immune signature based on 10 IGPs was constructed to stratify patients into low and high risk groups, exhibiting different clinical outcomes. A nomogram, combining immune signature, 1p/19q status, and tumor grade, was able to predict the overall survival (OS) with c-indices of 0.85, 0.80, 0.80, 0.79, and 0.75 in the training, internal validation, external validation, and tissue sample cohorts, respectively.

**Conclusions:**

This study was the first to report a comparative profiling of immune genes in large LGG cohorts. A promising individualized immune signature was developed to estimate the survival time for LGG patients.

## Introduction

Glioma is the most common and lethal brain tumor in the central nervous system^1^. According to the World Health Organization (WHO) grading system, gliomas are classified into grades I–IV based on their histological characteristics^2^. Recent studies have described grade II and III gliomas as lower grade gliomas (LGGs)^3–5^, which are less aggressive than grade IV glioblastomas (GBMs). Patients with LGG are recommended to receive surgical resection combined with radio- and/or chemotherapy. However, due to its invasive nature, complete resection of LGGs is almost impossible, and local recurrence occurs at variable intervals. In addition, a considerable subset of LGGs progress to GBM within months, while others remain stable for years. Prediction of LGG survival, ranging from 1–15 years, still remains a major challenge. The identification of subsets of patients at high risk for recurrence and death, who may benefit from additional systemic therapy, is therefore urgently needed.

In 2016, the WHO developed a glioma classification system based on the integration of multiple genotypic events (IDH mutations and 1p/19q co-deletions), highlighting the prognostic roles of specific molecular parameters. A growing number of studies have proposed gene expression signatures for survival stratification of patients with LGG. However, none of them has been incorporated into clinical practice due to numerous issues, including overfitting on small discovery datasets and lack of clinical validation. The clinical application of approaches based on gene expression is also hampered by the heterogeneities between datasets and technical bias due to the use of multiple measurement platforms. To develop a robust signature with higher chances of clinical application, novel methods are therefore required for data processing.

Immunotherapy is a promising treatment in multiple cancers, including gliomas. Rather than time-honored “immune privilege”, it is now clear that immunological disorders are involved in initiation and progression of gliomas. We previously reported that excessive immune response and disorganized immune microenvironment strongly contributed to the short survival of glioma patients^6–10^. In addition, the validity of prognostic prediction has been significantly enhanced by integrating clinicopathological and immunological features^11^. In this study, we reviewed the transcriptomic data of 865 LGGs to develop and validate a signature based on the differential profiles of immune gene pairs (IGPs). An individualized model integrating these immune signatures with clinical characteristics was constructed, to achieve an improved estimate of survival times in patients with LGG.

## Material and methods

### Patients

**Figure 1:**
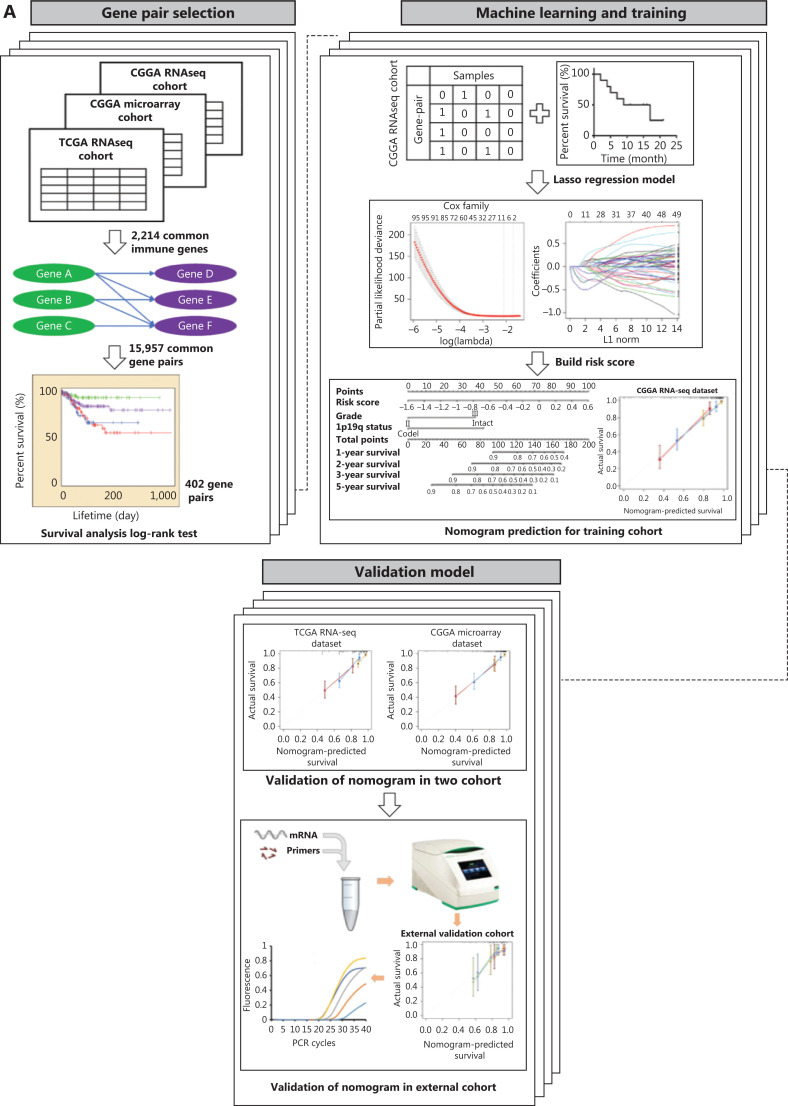

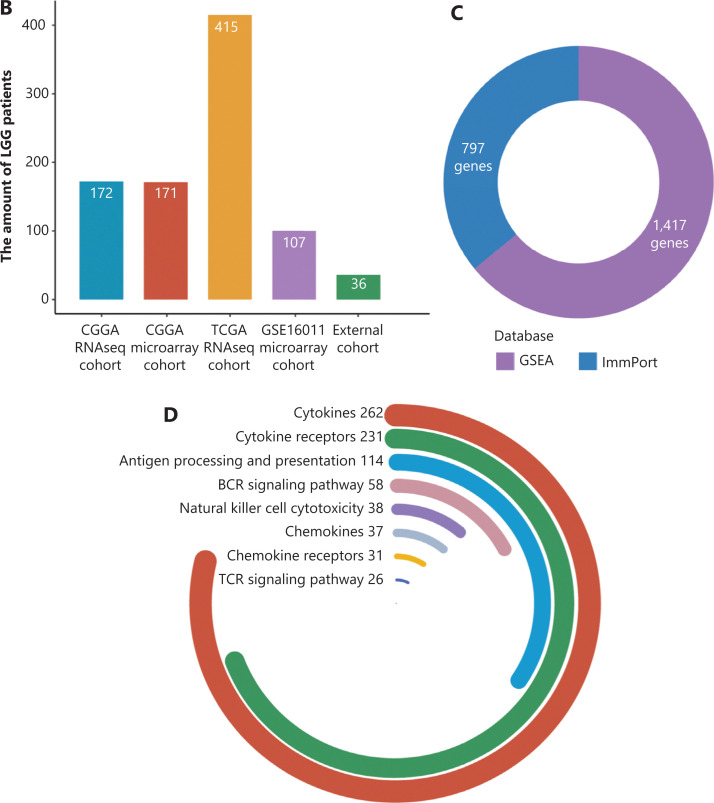
The study design. (A) Workflow graph of this study. (B) The histogram shows the number of patients collected from 4 cohorts. (C) A total of 1,417 and 797 immune-related genes were collected from the Gene Set Enrichment Analysis and ImmPort databases, respectively. (D) The immune-related genes from the ImmPort database were stratified into 7 categories, and the genes for cytokines, cytokine receptors, and antigen processing and presentation groups comprised the highest percentages of immune genes.

In this multiple cohort study, transcriptomics analysis was applied to LGG patients from 4 independent cohorts (**Figure 1A**). The expression data were collected from the Chinese Glioma Genome Atlas (CGGA, http://www.cgga.org.cn/index.jsp), The Cancer Genome Atlas (TCGA, http://cancergenome.nih.gov/) databases, and the Gene Expression Omnibus (GEO) database (https://www.ncbi.nlm.nih.gov/geo/query/acc.cgi?acc=gse16011). A total of 865 LGG patients were analyzed (**Figure 1B**). The in-house CGGA RNAseq and microarray cohorts were used for training and internal validation, respectively, and TCGA RNAseq and GSE16011 cohorts were analyzed for external validation. This study was approved by the Medical Ethics Committee of Beijing Tiantan Hospital (Approval No. KY2014-002-02).

### Tissue samples and molecular testing

The CGGA patients were treated by members of the CGGA group. Tumor tissue samples were collected at the time of surgery after obtaining informed consent. Neuropathologists established the diagnoses and ensured the quality of tissues for molecular testing. Overall survival (OS) was calculated from the date of diagnosis until death or the end of follow-up. The point of death was defined by death certification, which was obtained from local hospitals or police stations.

The tissue samples were immediately snap-frozen in liquid nitrogen after surgery. The percentage of tumor cells was assessed by hematoxylin and eosin staining, and the samples with more than 80% tumor cells were selected for RNA extraction. Total RNA from tumor samples was extracted by using the RNAprep pure Tissue Kit (Tiangen, Beijing, China) according to the manufacturer’s protocols. The concentration and optical density of RNA were measured using a NanoDrop ND-1000 spectrophotometer (NanoDrop Technologies, Wilmington, DE, USA).

The IDH1/2 mutation status was determined by pyrosequencing, as previously described^12^. Loss of 1p and 19q chromosome arms was inferred with a Gaussian window smoothing algorithm from RNA sequencing data, and exhibited a good concordance with the 1p/19q status determined by the SNP array (Mathews correlation coefficient = 0.94, *P* < 0.0001)^13^.

### Immune gene selection

A total of 2,879 immune-related genes were collected from the ImmPort database (https://immport.niaid.nih.gov) and Gene Set Enrichment Analysis (GSEA) dataset (http://software.broadinstitute.org/gsea/index.jsp). Among them, 2,214 immunological genes shared by three transcriptomics cohorts were reserved for further analyses (**Figure 1C**). The immunological genes from the ImmPort database were functionally categorized into 10 subsets (antigen processing and presentation, antimicrobials, B cell receptor signaling pathway, chemokines, chemokine receptors, cytokines, cytokine receptors, natural killer cell cytotoxicity, and the T cell receptor signaling pathway) (**Figure 1D**).

### Functional annotation and analysis

DAVID Bioinformatics Resources, version 6.8 (https://david.ncifcrf.gov/) was used to provide a comprehensive understanding of gene functions and biological processes. A false discovery rate less than 0.05 was regarded as statistically significant.

### Assessment of immune cell infiltration

The Microenvironment Cell Populations-counter method^14^ and CIBERSORT algorithm with LM22 gene signature, with 2 deconvolution methods, were used to provide highly precise quantitative information on the tumor microenvironment (TME) of cell contents in heterogeneous tissues, which allowed for sensitive and specific discrimination of the human immune cell and stromal cell phenotypes from transcriptome data, including B cells, T cells, natural killer cells, macrophages, dendritic cells, myeloid subsets, endothelial cells, and fibroblasts.

### Development of IGPs

Qualitative assessment is generally more reliable than quantitative assessment in differential gene expression analysis. Before developing the gene pairs, we arranged genes in the order of the initial letter. The IGPs were analyzed by pairwise comparisons of immune genes based on their expression values. Each IGP was viewed as an independent event with two possible outcomes [Gene(i) expression > Gene(j) expression or Gene(i) expression < Gene(j) expression]. Briefly, patients with the IGPs [Gene(i) expression > Gene(j) expression] were given a score of 1. Patients with IGPs [Gene(i) expression < Gene(j) expression] were given a score of 0. This was repeated for every immune gene pair to generate an IGP score for each patient.

### Real-time PCR (RT-PCR)

A total of 36 samples, including 13 WHO grade II gliomas (8 1p/19q intact and 5 co-deletion patients) and 23 WHO grade III gliomas (13 1p/19q intact and 10 co-deletion patients), were collected to assess the prediction accuracy and clinical usefulness of the nomogram model by real-time quantitative PCR. Specifically, 1 μg of total RNA was reverse-transcribed into cDNA using a RevertAid First Strand cDNA Synthesis Kit (Thermo Fisher Scientific, Waltham, MA, USA). The results of RT-PCR were normalized to the corresponding glyceraldehyde 3-phosphate dehydrogenase mRNA levels and the analyses were performed in triplicate to remove the outliers. Relative gene expression was determined using the 2^-dCt^ method. The primers were as follows:

CRH: forward: 5′-GGGAACCTCAACAAGAGCCC-3′, reverse: 5′-AACACGCGGAAAAAGTTGGC-3′

IFNB1: forward: 5′-GCTTGGATTCCTACAAAGAAGCA-3′, reverse: 5′-ATAGATGGTCAATGCGGCGTC-3′

HOXA9: forward: 5′-AAAAACAACCCAGCGAAGGC-3′, reverse: 5′-ACCGCTTTTTCCGAGTGGAG-3′

PRG3: forward: 5′-CAACTATCGCATTCAGTGCTGC-3′, reverse: 5′-GGGACCAGTAAGCAAAATTCCA-3′

IL10: forward: 5′-TCAAGGCGCATGTGAACTCC-3′, reverse: 5′-GATGTCAAACTCACTCATGGCT-3′

IL9: forward: 5′-CTCTGTTTGGGCATTCCCTCT-3′, reverse: 5′-GGGTATCTTGTTTGCATGGTGG-3′

PTH2: forward: 5′-GTAGGGGACTGTGCGGGAAG-3′, reverse: 5′-CTCCATCACCTGTGGAGAACC-3′

RETNLB: forward: 5′-AGCTCTCGTGTGCTAGTGTC-3′, reverse: 5′-TGAACATCCCACGAACCACA-3′

NKX2-5: FORWARD: 5′-CAAGTGTGCGTCTGCCTTTC-3′, reverse: 5′-CGCACAGCTCTTTCTTTTCGG-3′

PRLH: forward: 5′-TGCAAGTCGTACCCATCGG-3′, reverse: 5′-GGCGTACCAGGCAGGATTG-3′

NKX3-2: forward: 5′-ACCGAGACGCAGGTGAAAAT-3′, reverse: 5′-CACCTTTACGGCCACCTTCT-3′

UCN3: forward: 5′-GAGGCACCCGGTACAGATAC-3′, reverse: 5′-GAGGGACAGGGTGAACTTGG-3′

NR2C1: forward: 5′-CCAGATTGTGACAGCACTTGA-3′, reverse: 5′-CTTGGAGTAGAGCCGTCGT-3′

PRLHR: forward: 5′-TGAGTTCGGCCTGCTACAAC-3′, reverse: 5′-CCTGGCTAAGTGGCATCAGA-3′

REG1A: forward: 5′-ACCGGACCATCTCTCCAACT-3′, reverse: 5′-AGGGTTCCAAAGACTGGGGT-3′

TRIM31: forward: 5′-CGCAATCAGGTTCAACTCGC-3′, reverse: 5′-CTCGGGCATGTAGCCTCTTT-3′

PTX3: forward: 5′-CGAAATAGACAATGGACTCCATCC-3′, reverse: 5′-CTCATCTGCGAGTTCTCCAGCA-3.′

### Statistical analysis

All figures and statistical analyses were performed based on R for Windows, version 3.4.2 (http://www.r-project.org). The least absolute shrinkage and selection operator method from the glnmet package was used to reduce the overfitting. The risk-score formula for predicting survival was developed based on the 10 IGPs and the regression coefficient derived from Lasso regression analysis. The risk score for each patient was calculated as follows:

Risk score = (β*1*×score_(IGP1)_) + (β*2*×score_(IGP2)_) + (β*3*×score_(IGP3)_) + (β*4*×score_(IGP4)_) + (β*5*×score_(IGP5)_) + (β*6*×score_(IGP6)_) + (β*7*×score_(IGP7)_) + (β*8*×score_(IGP8)_) + (β*9*×score_(IGP9)_) + (β*10*×score_(IGP10)_)

Standard median splits can be used on either continuous or ordinal variables to convert them into dichotomous variables^15,16^. In the present study, patients were separated into low and high risk groups based on the cut-off point (median value). The Kaplan-Meier survival curve and log-rank test were used to evaluate the differences in OS. Univariate and multivariate Cox proportional hazard models were built using the Cox proportional hazards function from the survival package. All independent prognostic factors using multivariate analyses were then assessed by using the nomogram. The bootstrap method (*B* = 1,000) was performed to calculate the concordance index (C-index) for each independent dataset. *P* < 0.05 was considered as statistically significant.

## Results

### Identification of 402 prognostic IGPs

To identify the IGPs for model development, the following steps were performed. First, the 2,214 shared immune genes were examined by pairwise comparisons to generate 2,449,791 IGPs. Next, we excluded unevenly distributed IGPs (displaying over 95% of the scores equal to 1 or 0) from the 3 datasets. Then, a total of 15,957 overlapping IGPs were identified for further analyses. The prognostic values of the IGPs were evaluated using the log-rank test. There were 4,464, 4,911, and 3,557 prognostic IGPs in the CGGA RNAseq cohort (training), CGGA microarray cohort (internal validation), and TCGA RNAseq cohort (external validation), respectively. We found that there was a modest overlap of prognostic IGPs (*n* = 411) between these 3 cohorts. After removal of 9 controversial gene pairs, we obtained a list of 402 prognostic IGPs.

### Functional analysis of IGPs

**Figure 2:**
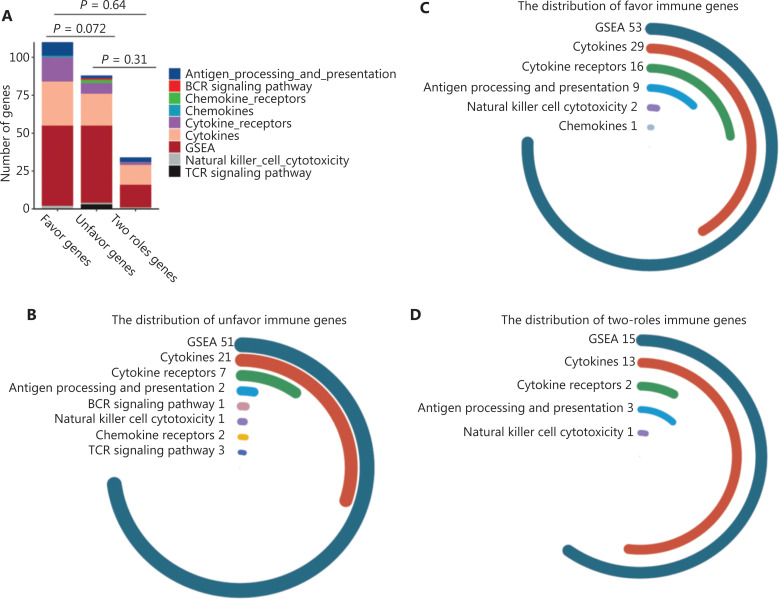

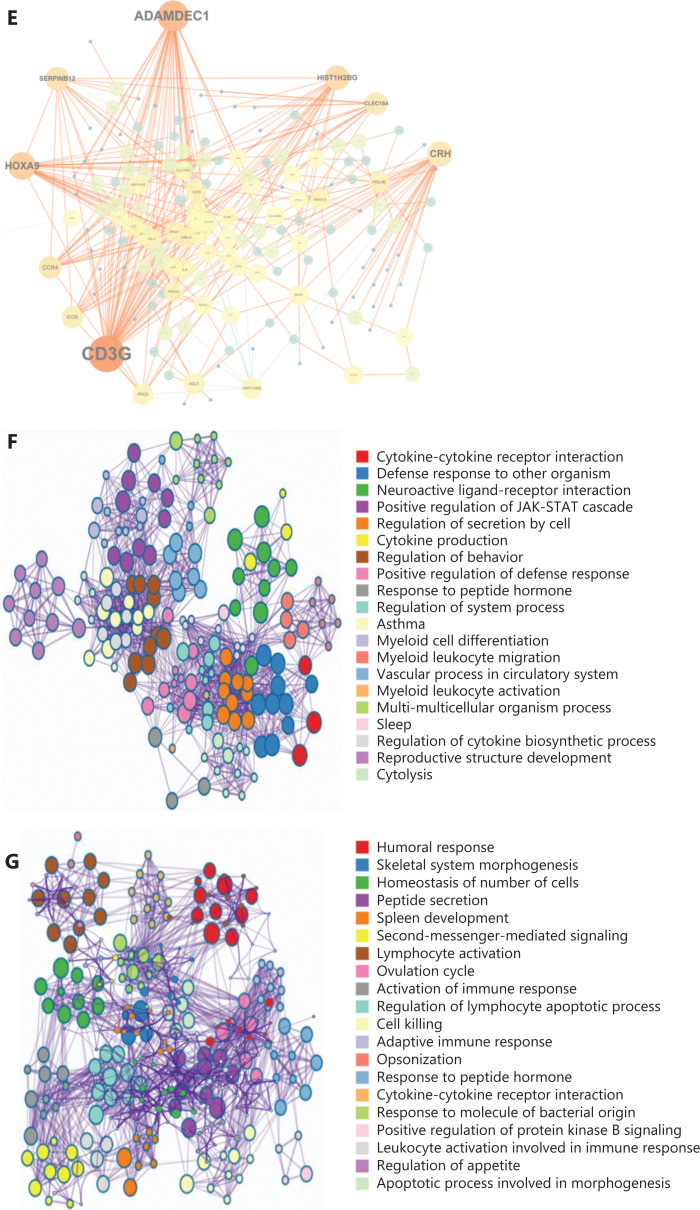
The landscape of 402 immune-related gene pairs (IPGs). (A) The 402 IPGs were composed of 110 favorable genes (FGs), 88 unfavorable genes (UGs), and 34, 2 role genes. (B–D) The distribution of immune genes from the GSEA and ImmPort databases for the FGs, UGs, and 2 role genes. (E) The gene co-expression network comprised of 232 unique immune genes in 402 immune-related gene pairs. The *CD3G*, *ADAMDEC1*, *HOXA9*, *HIST1H2BG*, *CCR4*, *CRH*, and *SERPINB12* genes showed the highest connection degrees in the network. Biological function analyses of FGs (F) and UGs (G).

To verify the prognostic utility of the selected IGPs, we defined IGPs with HR > 1 as unfavorable prognostic predictors (UPPs, *n* = 277), and the others as favorable prognostic predictors (FPPs, *n* = 125). The genes with higher expressions among UPPs or with lower expression among FPPs were identified as unfavorable genes (UGs, *n* = 88). In turn, the other genes were defined as favorable genes (FGs, *n* = 110). We found that FGs comprised a larger percentage of cytokine, cytokine receptor, and antigen processing genes (*P* = 0.072). Moreover, 34 genes behaving as both FG and UG among prognostic IGPs were termed two-side genes (TSGs) (**Figure 2A**). **Figure 2B and 2D** shows that FGs, UGs, and TSGs mainly included genes for cytokines, cytokine receptors, and proteins involved in antigen processing and presentation, as well as in natural killer cell cytotoxicity.

A gene co-expression network was constructed to evaluate the roles of IGPs. **Figure 2E** shows that the IGP co-expression network was represented by 232 unique immune genes. Of these, 7 genes with the highest connection degree were *CD3G*, *ADAMDEC1*, *HOXA9*, *HIST1H2BG*, *CCR4*, *CRH*, and *SERPINB12* (more than 15 connections/interactions). Among them, *CD3G* (*n* = 50), *ADAMDEC1* (*n* = 37), *HOXA9* (*n* = 28), HIST1H2BG (*n* = 20), and *CCR4* (*n* = 16) were core members of the UPP subset, accounting for 54.5% of the total 277 UPPs. However, *CRH* (*n* = 27) and *SERPINB12* (*n* = 18) were deemed as the core genes of the FPP subset. Further examination of the role of the above core genes suggested that *CD3G*, ADAMDEC1, *HOXA9*, *HIST1H2BG*, and *CCR4* were significantly overexpressed in GBM and associated with unfavorable prognoses, while the *CRH* had a protective function (**Supplementary Figure S1**).

We then used GO analysis to characterize the biological and functional annotations of the FGs and UGs. Because these subsets were related to immune functions, there were both similarities and differences with their GO annotations. **Figure 2F** shows that all FGs and UGs were significantly correlated with cytokine-cytokine receptor interactions and with responses to peptide hormones. In addition, FGs were strongly enriched in functions related to myeloid cell differentiation, myeloid leukocyte migration, myeloid leukocyte activation, and positive regulation of the JAK-STAT cascade. However, the UGs were mainly involved in lymphocyte regulation and physiological functions, including second-messenger-mediated signaling, skeletal system morphogenesis, and regulation of appetite (**Figure 2G**).

### The comparative score of core immune genes was a prognostic factor

**Figure 3:**
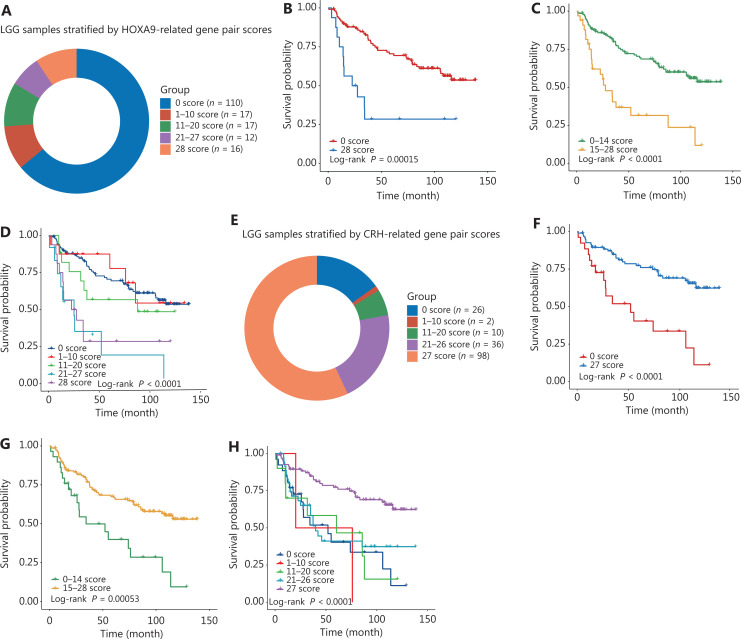
The accuracy of the hazard ratio (HR) scoring model in prognosis predictions. (A) The composition of different subgroups defined by the HR scores of *HOXA9*-RGs. (B) The Kaplan-Meier estimate showed the survival curves of patients in *HOXA9*-RGs in the 0 score and 28 score groups. (C) Similar to panel B, the Kaplan-Meier estimate showed the survival curves of patients in the low *HOXA9*-RGs score group (scores of 0−4) and high HOXA9-RGs score group (scores of 15−8). (D) The HR scores of *HOXA9*-RGs were separated by every 10 scores plus the minimum and maximum scores. The Kaplan-Meier curves of patients in 5 scored cohorts. (E–H) The CRH-RGs scoring model for predicting the overall survival of lower grade gliomas.

To determine the prognostic accuracy of the differential expression profiles, we developed an HR scoring algorithm to calculate the comparative pattern of specific genes. The *HOXA9*-related IGPs were the third highest number of unfavorable gene pairs in 402 prognostic IGPs. There was a total of 28 HOXA9-related IGPs. The HR score of *HOXA9*-related IGPs ranged from 0–28 for each patient. **Figure 3A** shows that the majority of LGG patients had a *HOXA9*-score of 0. Survival analysis revealed that these patients had a better prognosis than those with a *HOXA9*-score of 28 (**Figure 3B**, *P* = 0.00015). When strata were considered based on the 0–14 score cohorts and 15–28 score cohorts, patients in the higher *HOXA9*-score cohorts exhibited a significantly reduced OS compared to those in the lower HOXA9-score cohorts (**Figure 3C**, *P* < 0.0001). Furthermore, when patients were divided into 5 groups based the HOXA9-score, patient survival tended to decrease with an increase in the *HOXA9*-score (**Figure 3D**, *P* < 0.0001).

The *CRH*-related IGPs were the most frequently favorable gene pairs in 402 prognostic IGPs. There was a total of 27 *CRH*-related IGPs. The HR score of *CRH*-related IGPs ranged from 0–27 for each patient. Similar to the *HOXA9*-RGPs prediction model, risk prediction on the FPP *CRH*-score resulted in a significant stratification of OS. Specifically, patients with a *CRH*-score of 27 had a better prognosis than those with a *CRH*-score of 0 (**Figure 3E and 3F**, *P* < 0.0001). When patients were classified into 2 or 5 groups based on the *CRH*-score, a robust inverse correlation was observed between the *CRH*-score and survival time (**Figure 3G and 3H**). These results showed that the gene pair patterns of core immune genes enhanced our understanding of gene relationships and provided valuable information for the management of glioma patients.

### Prognostic significance of the immune signature in the discovery and validation cohorts

**Figure 4:**
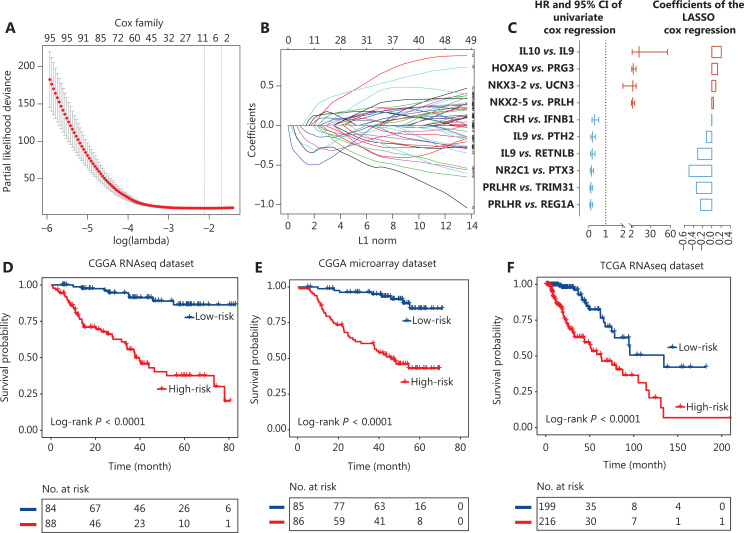
Identification of the 10 immune-related gene pair (IGP) signature for lower grade glioma patients. (A) One-thousand times cross-validation for tuning parameter selection in the LASSO Cox regression model. (B) LASSO coefficient profiles of 10 IGPs by the largest value of lambda (1se). (C) The hazard ratios and the 95% confidence intervals using univariate analyses, and the values of coefficients using Lasso analyses of 10 IGPs. The dichotomized immune signature risk score allowed the segmentation of patients into high and low risk groups in the Chinese Glioma Genome Atlas (CGGA) RNAseq cohort (D), CGGA microarray cohort (E), and The Cancer Genome Atlas RNAseq cohort (F).

To generate an immune signature with prognostic value, we conducted LASSO regression based on the 402 prognostic IGPs to minimize the risk of overfitting (**Figure 4A and 4B**). Ten IGPs, including 4 UPPs (*HOXA9-PRG3*, *NKX2-5-PRLH*, IL10-IL9, *NKX3-2-UCN3*) and 6 FPPs (*CRH-IFNB1*, *IL9-PTH2*, *IL9-RETNLB*, *NR2C1-PTX3*, *PRLHR-REG1A*, and *PRLHR-TRIM31*), were selected using LASSO regression analysis (**Figure 4C**).

To determine the prognostic value of the immune signature, the median risk value was defined as the cutoff for patient classification into high or low risk groups. We found that patients in the low risk group (median survival: undefined) had a significantly longer survival time than patients in the high risk group [median survival: 37.76 months; hazard ratio (HR), 7.58; 95% confidence interval (CI), 4.31–13.31, *P* < 0.0001] (**Figure 4D**). The same formula was used for the internal and external validation cohorts to further verify the prognostic utility of the immune signature. Patients were also divided into low and high risk groups according to the cutoff value. As expected, patients in the low risk group had a longer survival time than patients in the high risk group (CGGA microarray cohort, median survival, undefined *vs.* 45.8 months. HR, 7.02; 95% CI, 4.04–12.22, *P* < 0.0001; TCGA cohort, median survival, 134.18 months *vs.* 62.91 months; HR, 3.51; 95% CI, 2.26–5.48, *P* < 0.0001) (**Figure 4E and 4F**). The results indicated that the identified immune signature had robust prognostic value across different testing platforms.

Next, patients were stratified based on clinicopathological features, including WHO grade (II and III), 1p/19q status (co-deletion and intact), IDH status (mutation and wild-type), and histology (astrocytoma and oligoastrocytoma). In the CGGA RNAseq cohort, the immune signature was an unfavorable indicator in all stratified analyses (**Supplementary Figure S2**) and patients in the low risk group presented a better prognosis than those in the high risk group. Furthermore, the high risk group tended to present a poor outcome in subgroups of the CGGA microarray (**Supplementary Figure S3**) and TCGA RNAseq (**Supplementary Figure S4**) cohorts, even if some of the results were statistically marginally significant.

In addition, uni- and multivariate Cox regression analyses revealed that the immune signature was an independent prognostic factor for LGG patients in 3 independent cohorts after adjusting for age, sex, WHO grade, IDH status, and 1p/19q status (**Supplementary Table S1**). Moreover, HR analysis showed that the presence of the immune signature was a significant detrimental factor in nearly all subgroups (**Supplementary Figure S5**). Therefore, the immune signature was a robust predictor of OS in LGG patients.

### Association of the immune signature with tumor immune infiltration

**Figure 5:**
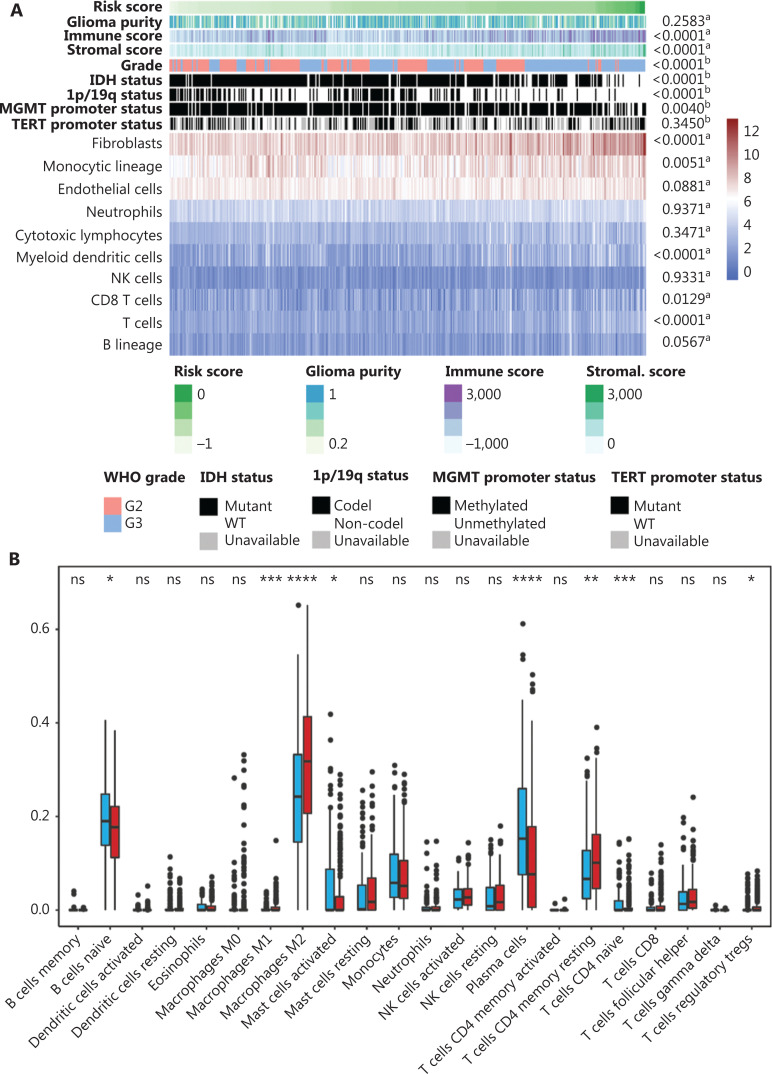

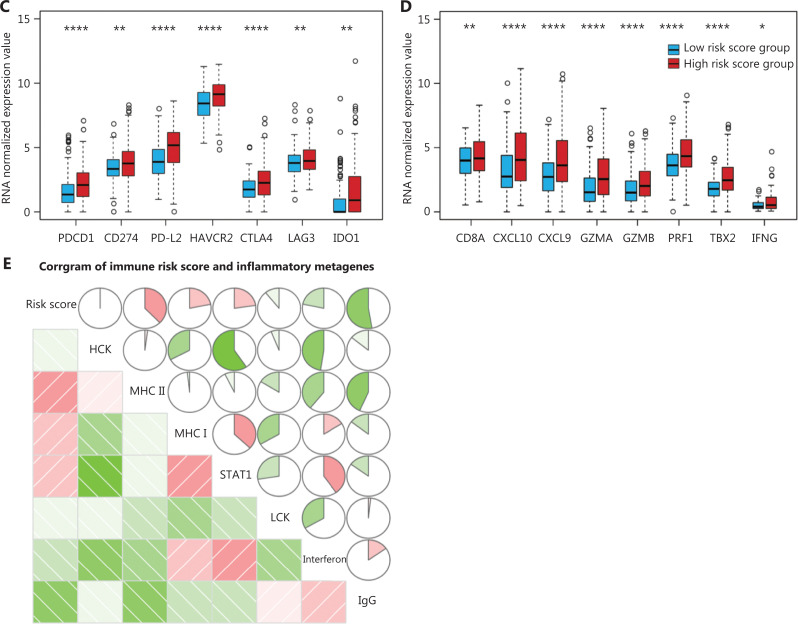
The association between immune signature and tumor immune infiltration characteristics. (A) The landscape of clinical, molecular features, and tumor microenvironment (TME) cells together with immune risk score (a, The association between risk score and continuous variables was assessed using Pearson’s correlation tests. b, The distribution of risk score between 2 groups was assessed using one-way analysis of variance). (B) The distribution characteristics of TME cells between low and high immune risk score groups. The scattered dots represent TME cell values and the thick lines represent the median value (*,**, ***, and **** represent *P* < 0.05, *P* < 0.01, *P* < 0.001, and *P* < 0.0001, respectively). (C) The normalized expression value of immune activation-relevant genes in the 2 groups. (D) The normalized expression values of immune checkpoint-relevant genes in the 2 groups. (E) The relationship between immune risk scores and T-cell-related metagenes.

To characterize the immune risk score, we analyzed the association between the risk score and clinico-pathological and tumor-related immune parameters. The TME composition, composed of glioma purity, and human immune cells and stromal cells, was deconvoluted using the ESIMATE method, MCPcounter, and CIBERSORT algorithm. The glioma samples were arranged in order of increasing risk scores (**Figure 5A**). Pearson’s correlation analysis showed that the risk score had a significant positive association with the immune and stromal scores. Patients in the high immune risk score group mainly exhibited higher infiltration of M2-polarized TAMs and T cell CD4 resting memory. The infiltration of naïve B cells, activated mast cells, and plasma cells were significantly higher in the lower immune risk group (**Figure 5B**). In terms of clinical characteristics, we found that grade II glioma patients, as well as patients with the IDH mutation, 1p/19q codel, and methylated MGMT promoter were more likely to be in the low immune risk score group (**Figure 5A**).

Moreover, the cytokine and chemokine environments characterizing the low and high immune risk score groups were analyzed^17^. The *IDO1*, *CD274*, *HAVCR2*, *PDCD1*, *CTLA4*, *LAG3*, and *PD-L2* genes were identified as immune checkpoint-relevant transcripts^9^ (**Figure 5C**). The *CXCL10*, *CXCL9*, *GZMA*, *GZMB*, *PRF1*, *CD8A*, *IFNG,* and *TBX2* genes were identified as immune activation-related transcripts (**Figure 5D**). The high immune risk score group exhibited a higher expression of immune checkpoint-related genes, while the immune activation-related genes displayed a relatively poor expression in the low immune risk score group. To gain a deeper understanding of the relationships between inflammation and the immune risk scores, 7 lymphocyte-specific metagenes, including 7 subtypes of immune inflammation response genes, were collected^18^. **Figure 5E** shows that the activities of *MHC I*, *MHC II*, and *STAT1* were specifically elevated in the high immune risk score group. Together, the results indicated that gliomas with higher immune risk scores were more likely to possess a more complex physiological immune homeostasis.

### Development and validation of a prognostic nomogram

**Figure 6:**
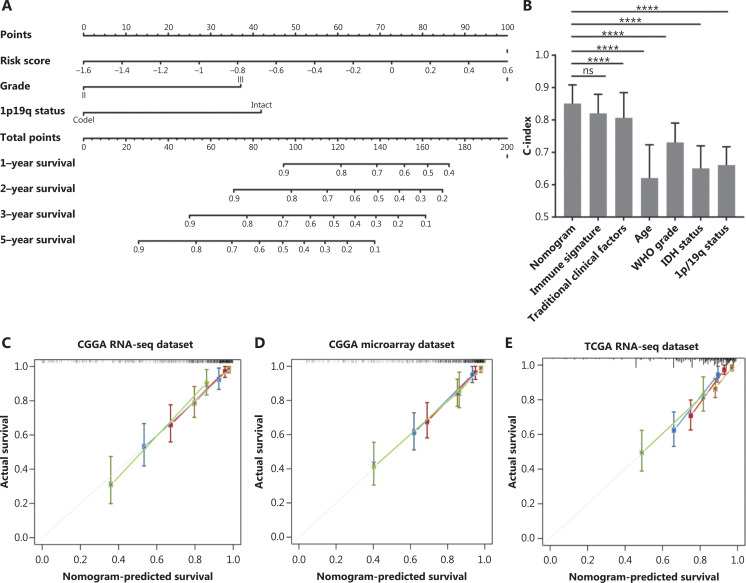

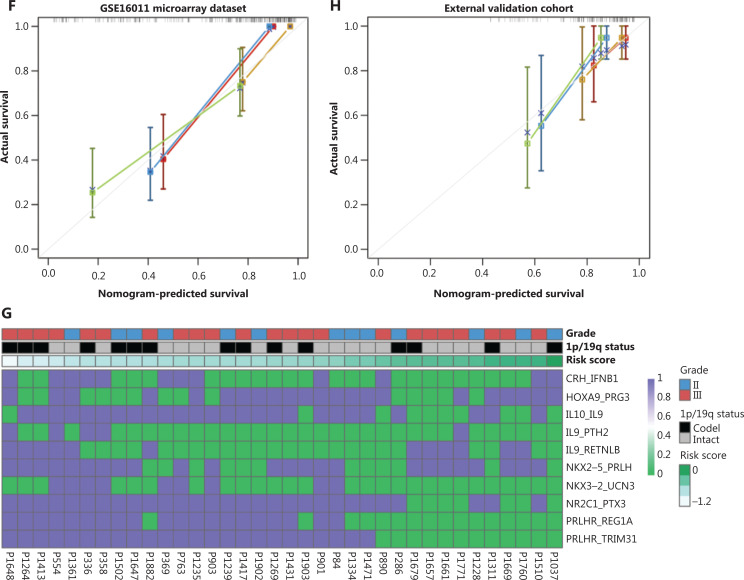
The performance of the nomogram in the training and validation cohorts. (A) The nomogram for predicting 1-year, 2-year, 3-year, and 5-year overall survivals for lower grad glioma (LGG) patients. (B) The c-index in predicting overall survival (OS) was compared between the nomogram model and other factors, including immune signature, age, World Health Organization grade, IDH status, and 1p/19q status in the Chinese Glioma Genome Atlas RNAseq cohort (mean ± SD; ****P < 0.0001, Student’s *t*-test). The calibration curve for predicting the OS for LGG patients in training (C), internal validation (D). and external validation (E–F); goldenrod: 1-year survival, firebrick: 2-year survival, steel blue: 3-year survival, and dark olive green: 5-year survival. (G) The heat map shows the 10 immune-related gene pair profiles with clinicopathology information of 36 frozen tissue LGG samples. (H) The calibration curve for predicting the OS for the 36 patients.

To maximize the predictive accuracy, a nomogram combining our immune signature with traditional clinical features was developed. Three independent covariates (immune signature, 1p/19q status, and grade) were selected using multivariate Cox regression analysis (**Figure 6A**). The nomogram was able to predict OS with a c-index of 0.85, which was significantly higher than those obtained with the individual traditional clinical and the combined traditional clinical factors (**Figure 6B**). The calibration plots indicated an optimal agreement between the predictions and the observed 1, 2, 3, and 5-year survival percentages (**Figure 6C**).

To test the universality of the identified immune signature with respect to other populations and platforms, the nomogram was applied to validation cohorts, yielding c-indices of 0.80 in the CGGA microarray cohort (internal validation), 0.80 in TCGA RNAseq cohort (external validation), and 0.79 in the GSE16011 microarray cohort (independent validation). The predicted survival times based on the nomogram were highly consistent with the actual survival data from the validation tests (**Figure 6D, 6F and Supplementary Figure S6**).

We also collected independent tumor tissue samples, including 36 LGG patients, for immune signature validation using RT-PCR (**Figure 6G**). The nomogram confirmed its predictive robustness in this cohort, with a c-index of 0.75 (**Figure 6H**). These results showed that the nomogram was accurate in predicting patient survival, and proved to be a new potential tool for use in clinical practice.

## Discussion

Accounting for approximately one-third of adult gliomas, LGGs show highly variable clinical behaviors. Subgroups of LGG exhibit poor outcomes, similar to GBM, in spite of substantial differences in histology and genetic background^19^. Discrimination of patients at high risk of death, who might benefit from additional intensive treatment, remains one of the major clinical challenges. Although great effort has been expended in the identification of gene expression-based prognostic signatures, their clinical application is still limited.

In this study, we reviewed gene expression profiles from different populations and designed a robust transcriptomic comparison method based on microarray, RNAseq, and routine qPCR technologies. We characterized the prognostic profiles of gene pairs, which resulted in an immune signature that allowed for improved estimation of LGG patient survival. Conventional methods based on differentially-expressed genes are of limited applicability due to differences of the sequencing platform and batch effects. The gene-pair comparison method minimizes the impact of the batch effect, using differentially-expressed genes at the individual level.

It has been well-established that innate and adaptive immune systems promote glioma malignancy by multiple mechanisms^20–22^. Recent studies proposed immunotherapy as a promising strategy for glioma^23–25^. In comparison with GBM, LGG displays moderate immune-mediated reactions characterized by weakened local immune response and lower immune cell infiltration^26^. We previously reported an immune gene signature for GBM, suggesting that the estimation of immune genes was a valuable strategy for prognosis and prediction of treatment effectiveness^27^. However, few immunological signatures have been established for LGG. Here, an immune signature was established based on pair-wise comparisons. Unlike traditional methods for the normalization of gene expression data, this approach was based on comparative gene expressions, which provided more reliable results, avoiding the heterogeneity between data sets and technical bias related to the diversity of measurement platforms. Therefore, this signature exhibited a robust predictive performance across different platforms and populations.

A total of 402 IGPs, including 277 UPPs and 125 FPPs, exhibited high prognostic consistency for LGG across 3 distinct datasets. The GO analyses revealed that FG-related immune functions, such as myeloid leukocyte differentiation, activation, and migration were closely correlated with the promotion of an anti-tumor immune responses and with a favorable prognosis for LGG patients. *HOXA9* and *CRH* were core genes, which were more frequent among the IGPs. HOXA9 is a homeodomain-containing transcription factor, predicting poor survival in patients with leukemia^28^, ovarian cancer^29^ and breast cancer^30^. CRH is a peptide hormone involved in the response of stress and inflammation^31,32^. The role of these factors in other cancers is consistent with our findings, which further confirmed the potential suitability of *HOXA9* and *CRH* status as predictors of clinical outcome. Moreover, our HR scoring system proved an excellent predictor of survival, providing a model for risk stratification based on core gene comparative profiles.

In this study, an immune signature was developed based on the profiling of 402 IGPs. After reducing the overfitting of the gene pairs profile by the LASSO regression model, a risk signature for glioma was developed, based on 10 comparative gene pairs. IL10 was the one of the key interleukins composed of unfavorable IGPs. Interleukin 10 encoded by the *IL10* gene, is an anti-inflammation cytokine^33^. IL10 is involved in ERK1/2, p38, and NF-kB signaling, and down-regulates co-stimulatory molecules on macrophages^34^. Macrophages are key cells in the innate immune system. They phagocytose pathogens and cellular debris, promote inflammation, and have important roles in tumor immunity^35–37^. Depending on the microenvironment, macrophages can polarize to M1 (inflammatory) or M2 (anti-inflammatory) phenotypes. Rafael et al.^38,39^ found that the IL-10/IL-10R axis was required for polarization of microglia to the M2-like phenotype, and promoteed tumor growth in an IL-10-dependent manner. In addition, M2-polarized TAMs promoted tumorigenesis, the epithelial-mesenchymal transition, proliferation, and infiltration through the IL10-related signaling pathway in cancers^40–42^.

Because of the robust relationship between genes, the immune signature retained its prognostic significance in 3 large LGG cohorts tested by RNAseq or microarray platforms. Furthermore, the immune signature, either as a continuous or categorical variable, was an independent prognostic factor, after adjustment for clinical and molecular characteristics. These findings highlighted the potential of the immune signature as a new tool to improve prognostic accuracy.

Based on the analysis of the relationship between the immune signature and the TME, we found that patients in the high risk group had a high number of specific immune cell populations (T cells, CD8+ T cells, cytolytic lymphocytes, and monocytic lineage), and exhibited elevated expressions of genes involved in T-cell activation (*CXCL10*, *CXCL9*, *GZMA*, *GZMB*, *PRF1*, and *TBX2*), antigen presentation (*MHC I* and *MHC II*), and interferon signal transduction (*STAT1*). In addition, the immune checkpoints (*PDCD1*, *CD274*, *PD-L2*, *HAVCR2*, *CTLA4*, *LAG3*, and *IDO1*), which block T cell activation, were significantly upregulated in patients from the high risk group. This observation indicated that patients with a high immune risk score tended to have more complex interactions between gliomas and their immunological microenvironments.

Although numerous valuable prognostic factors are known, the appropriate combination of markers for individualized prognostic predictions is still needed. Nomograms are graphical depictions of predictive statistical models, with a proven advantage over traditional systems for individualized prediction, and have been developed for various types of cancers^43^. To date, the assessment of nomograms has been rarely conducted for LGG patients. Here, we developed a nomogram prediction module by integrating the identified immune signatures with routine clinical features to predict 1-year, 2-year, 3-year, and 5-year survival in LGG patients. The nomogram module exhibited more accurate projections in predicting OS than other prognosis markers reported in previous studies^44^.

We recognize some limitations in this study. First, some important molecular markers, including TERT, MGMT, CDKN2A/B, were not collected from TCGA, CGGA, and GSE16011 cohorts. Second, the GSE16011 and TCGA cohorts did not provide information of the extents of surgical resections. Third, there was some missing data of the 1p/19q status and IDH status in the GSE16011 cohort.

To our knowledge, this nomogram was the first to apply a gene comparison approach to gliomas, and exhibited excellent predictive accuracy across RNAseq, microarray, and qPCR cohorts. The nomogram provided an individualized and comprehensive estimate of the prognostic risks and may substantially contribute to clinical management decisions.

## Conclusions

In the present study, we first profiled the comparative pattern of immune genes and identified an immune signature based on 10 gene pairs with independent prognostic values of LGG patients. We established an individualized prognostic model combining the immune signatures with clinical information. Our study possessed good versatility and efficiency, large RNA sequencing cohorts for discovery and validation, and a robust validity based on RT-PCR. However, our study was limited due to its retrospective nature and should be validated by future studies. Overall, our findings provided clues to estimate the survival of LGG patients, and has great promise for the identification of novel molecular targets.

## Data Availability

The expression data were collected from the Chinese Glioma Genome Atlas (CGGA, http://www.cgga.org.cn/index.jsp), The Cancer Genome Atlas (TCGA, (http://cancergenome.nih.gov/) database, and the Gene Expression Omnibus database (https://www.ncbi.nlm.nih.gov/geo/query/acc.cgi?acc=gse16011).

## References

[1] Ostrom QT, Gittleman H, Farah P, Ondracek A, Chen Y, Wolinsky Y (2013). CBTRUS statistical report: primary brain and central nervous system tumors diagnosed in the united states in 2006-2010. Neuro Oncol.

[2] Louis DN, Ohgaki H, Wiestler OD, Cavenee WK, Burger PC, Jouvet A (2007). The 2007 WHO classification of tumours of the central nervous system. Acta Neuropathol.

[3] Suzuki H, Aoki K, Chiba K, Sato Y, Shiozawa Y, Shiraishi Y (2015). Mutational landscape and clonal architecture in grade II and III gliomas. Nat Genet.

[4] Eckel-Passow JE, Lachance DH, Molinaro AM, Walsh KM, Decker PA, Sicotte H (2015). Glioma groups based on 1p/19q, IDH, and TERT promoter mutations in tumors. N Engl J Med.

[5] Brat DJ, Verhaak RG, Aldape KD, Yung WK, Salama SR, Cooper LA (2015). Comprehensive, integrative genomic analysis of diffuse lower-grade gliomas. N Engl J Med.

[6] Zhang C, Cheng W, Ren X, Wang Z, Liu X, Li G (2017). Tumor purity as an underlying key factor in glioma. Clin Cancer Res.

[7] Chen Q, Han B, Meng X, Duan C, Yang C, Wu Z (2019). Immunogenomic analysis reveals LGALS1 contributes to the immune heterogeneity and immunosuppression in glioma. Int J Cancer.

[8] Cai J, Chen Q, Cui Y, Dong J, Chen M, Wu P (2018). Immune heterogeneity and clinicopathologic characterization of IGFBP2 in 2447 glioma samples. Oncoimmunology.

[9] Wu P, Geng B, Chen Q, Zhao E, Liu J, Sun C (2020). Tumor cell-derived TGFbeta1 attenuates antitumor immune activity of T cells via regulation of PD-1 mRNA. Cancer Immunol Res.

[10] Zha C, Meng X, Li L, Mi S, Qian D, Li Z (2020). Neutrophil extracellular traps mediate the crosstalk between glioma progression and the tumor microenvironment via the HMGB1/RAGE/IL-8 axis. Cancer Biol Med.

[11] Qiu H, Li Y, Cheng S, Li J, He C, Li J (2020). A prognostic microenvironment-related immune signature via estimate (promise model) predicts overall survival of patients with glioma. Front Oncol.

[12] Yan W, Zhang W, You G, Zhang J, Han L, Bao Z (2012). Molecular classification of gliomas based on whole genome gene expression: a systematic report of 225 samples from the chinese glioma cooperative group. Neuro Oncol.

[13] Wang ZL, Zhao Z, Wang Z, Zhang CB, Jiang T (2019). Predicting chromosome 1p/19q codeletion by RNA expression profile: a comparison of current prediction models. Aging (Albany NY).

[14] Becht E, Giraldo NA, Lacroix L, Buttard B, Elarouci N, Petitprez F (2016). Estimating the population abundance of tissue-infiltrating immune and stromal cell populations using gene expression. Genome Biol.

[15] Garcia D, MacDonald S, Archer T (2015). Two different approaches to the affective profiles model: median splits (variable-oriented) and cluster analysis (person-oriented). PeerJ.

[16] DeCoster J, Gallucci M, Iselin AMR (2011). Best practices for using median splits, artificial categorization, and their continuous alternatives. J Exp Psychopathol.

[17] Zeng D, Li M, Zhou R, Zhang J, Sun H, Shi M (2019). Tumor microenvironment characterization in gastric cancer identifies prognostic and immunotherapeutically relevant gene signatures. Cancer Immunol Res.

[18] Rody A, Holtrich U, Pusztai L, Liedtke C, Gaetje R, Ruckhaeberle E (2009). T-cell metagene predicts a favorable prognosis in estrogen receptor-negative and HER2-positive breast cancers. Breast Cancer Res.

[19] Wang Z, Hao Y, Zhang C, Wang Z, Liu X, Li G (2017). The landscape of viral expression reveals clinically relevant viruses with potential capability of promoting malignancy in lower-grade glioma. Clin Cancer Res.

[20] Ousman SS, Kubes P (2012). Immune surveillance in the central nervous system. Nat Neurosci.

[21] Fridman WH, Zitvogel L, Sautes-Fridman C, Kroemer G (2017). The immune contexture in cancer prognosis and treatment. Nat Rev Clin Oncol.

[22] Muldoon LL, Alvarez JI, Begley DJ, Boado RJ, Del Zoppo GJ, Doolittle ND (2013). Immunologic privilege in the central nervous system and the blood-brain barrier. J Cereb Blood Flow Metab.

[23] Brown CE, Alizadeh D, Starr R, Weng L, Wagner JR, Naranjo A (2016). Regression of glioblastoma after chimeric antigen receptor T-cell therapy. N Engl J Med.

[24] He Y, Rivard CJ, Rozeboom L, Yu H, Ellison K, Kowalewski A (2016). Lymphocyte-activation gene-3, an important immune checkpoint in cancer. Cancer Sci.

[25] Parry RV, Chemnitz JM, Frauwirth KA, Lanfranco AR, Braunstein I, Kobayashi SV (2005). CTLA-4 and PD-1 receptors inhibit t-cell activation by distinct mechanisms. Mol Cell Biol.

[26] Han S, Zhang C, Li Q, Dong J, Liu Y, Huang Y (2014). Tumour-infiltrating CD4(+) and CD8(+) lymphocytes as predictors of clinical outcome in glioma. Br J Cancer.

[27] Cheng W, Ren X, Zhang C, Cai J, Liu Y, Han S (2016). Bioinformatic profiling identifies an immune-related risk signature for glioblastoma. Neurology.

[28] Collins CT, Hess JL (2016). Role of HOXA9 in leukemia: dysregulation, cofactors and essential targets. Oncogene.

[29] Ko SY, Ladanyi A, Lengyel E, Naora H (2014). Expression of the homeobox gene HOXA9 in ovarian cancer induces peritoneal macrophages to acquire an M2 tumor-promoting phenotype. Am J Pathol.

[30] Gilbert PM, Mouw JK, Unger MA, Lakins JN, Gbegnon MK, Clemmer VB (2010). HOXA9 regulates BRCA1 expression to modulate human breast tumor phenotype. J Clin Invest.

[31] Quintanar JL, Guzman-Soto I (2013). Hypothalamic neurohormones and immune responses. Front Integr Neurosci.

[32] Bethin KE, Vogt SK, Muglia LJ (2000). Interleukin-6 is an essential, corticotropin-releasing hormone-independent stimulator of the adrenal axis during immune system activation. Proc Natl Acad Sci U S A.

[33] Batchu RB, Gruzdyn OV, Kolli BK, Dachepalli R, Umar PS, Rai SK (2021). IL-10 signaling in the tumor microenvironment of ovarian cancer. Adv Exp Med Biol.

[34] Said EA, Dupuy FP, Trautmann L, Zhang Y, Shi Y, El-Far M (2010). Programmed death-1-induced interleukin-10 production by monocytes impairs CD4+ t cell activation during hiv infection. Nat Med.

[35] Cai J, Zhang W, Yang P, Wang Y, Li M, Zhang C (2015). Identification of a 6-cytokine prognostic signature in patients with primary glioblastoma harboring M2 microglia/macrophage phenotype relevance. PLoS One.

[36] Henze AT, Mazzone M (2016). The impact of hypoxia on tumor-associated macrophages. J Clin Invest.

[37] Meng X, Duan C, Pang H, Chen Q, Han B, Zha C (2019). DNA damage repair alterations modulate M2 polarization of microglia to remodel the tumor microenvironment via the p53-mediated MDK expression in glioma. EBioMedicine.

[38] Lopes RL, Borges TJ, Zanin RF, Bonorino C (2016). IL-10 is required for polarization of macrophages to M2-like phenotype by mycobacterial Dnak (heat shock protein 70). Cytokine.

[39] Laffer B, Bauer D, Wasmuth S, Busch M, Jalilvand TV, Thanos S (2019). Loss of IL-10 promotes differentiation of microglia to a M1 phenotype. Front Cell Neurosci.

[40] Yang L, Dong Y, Li Y, Wang D, Liu S, Wang D (2019). IL-10 derived from M2 macrophage promotes cancer stemness via JAK1/STAT1/NF-kappaB/Notch1 pathway in non-small cell lung cancer. Int J Cancer.

[41] Liu CY, Xu JY, Shi XY, Huang W, Ruan TY, Xie P (2013). M2-polarized tumor-associated macrophages promoted epithelial-mesenchymal transition in pancreatic cancer cells, partially through TLR4/IL-10 signaling pathway. Lab Invest.

[42] Qi L, Yu H, Zhang Y, Zhao D, Lv P, Zhong Y (2016). IL-10 secreted by M2 macrophage promoted tumorigenesis through interaction with JAK2 in glioma. Oncotarget.

[43] Balachandran VP, Gonen M, Smith JJ, DeMatteo RP (2015). Nomograms in oncology: more than meets the eye. Lancet Oncol.

[44] Cheng W, Zhang C, Ren X, Wang Z, Liu X, Han S (2017). Treatment strategy and IDH status improve nomogram validity in newly diagnosed GBM patients. Neuro-Oncol.

