# Supplementary material for: Comparative profiling of immune genes improves the prognoses of lower grade gliomas
Source: Cancer Biol Med. 2021 Oct 9;19(4):533–50. doi: 10.20892/j.issn.2095-3941.2021.0173 (PMC9088193; doi:10.20892/j.issn.2095-3941.2021.0173)
Supplement: Supplementary file 1 [file cbm-19-533-s001.pdf]

Supplementary materials

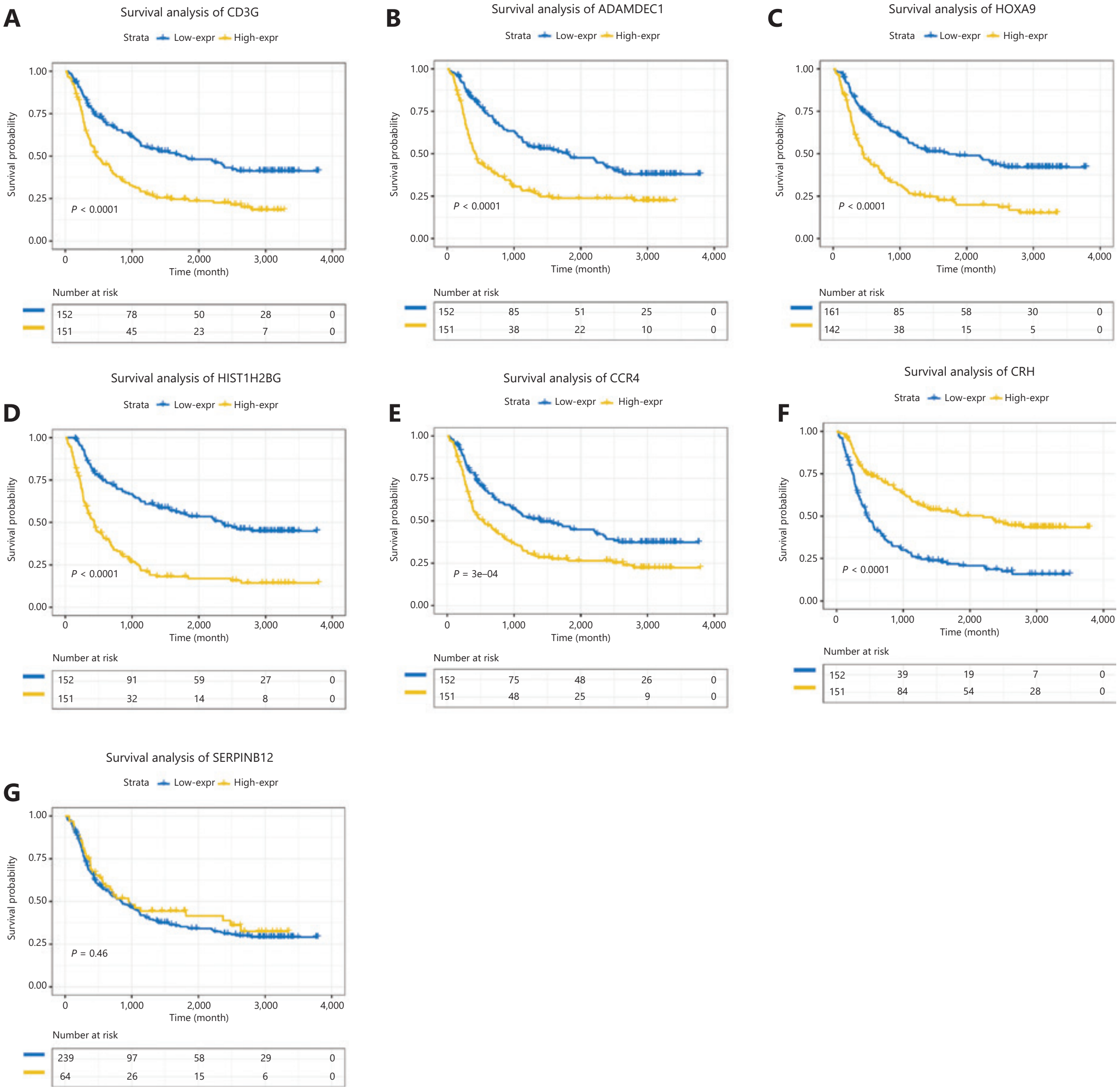

Figure S1 The survival analysis of 7 core genes in 402 immune-related gene pairs. (A–E) Kaplan-Meier analyses of overall survival based on five UGs, including CD3G, ADAMDEC1, HOXA9, HIST1H2BG and CCR4. (F, G) Kaplan-Meier analysis of overall survival based on two FGs, including CRH and SERPINB12.

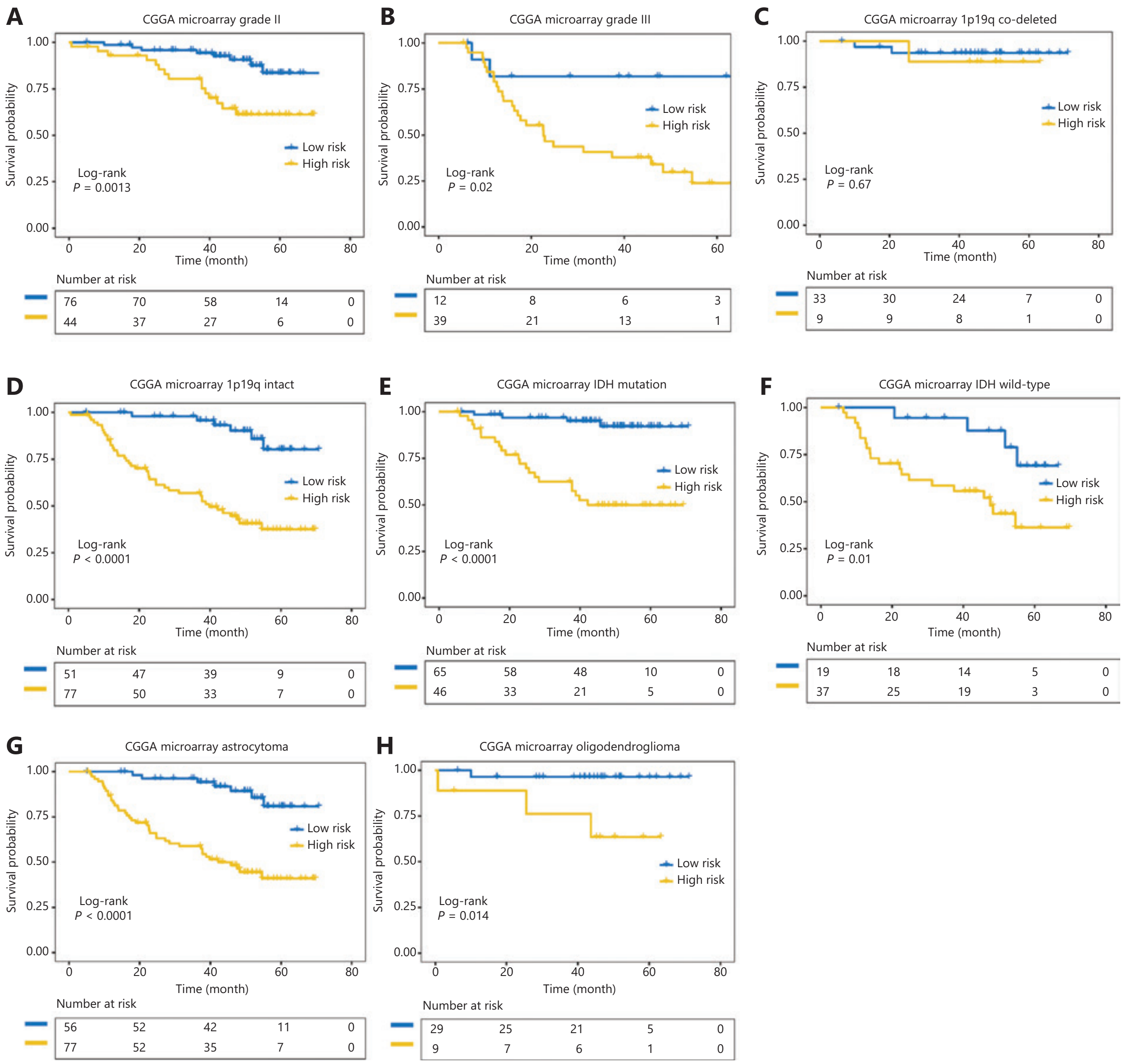

Figure S2 The clinical significance of 10 IGPs signature for LGG patients in CGGA RNAseq cohort. The Kaplan-Meier survival curves for LGG patients with grade II (A), grade III (B), 1p/19q codeletion (C), 1p/19q intact (D), IDH mutation (E), IDH wildtype (F), astrocytoma (G) and oligodendroglioma (H). Patients were divided into lower and higher risk score groups and *P*-value was the result of log-rank test.

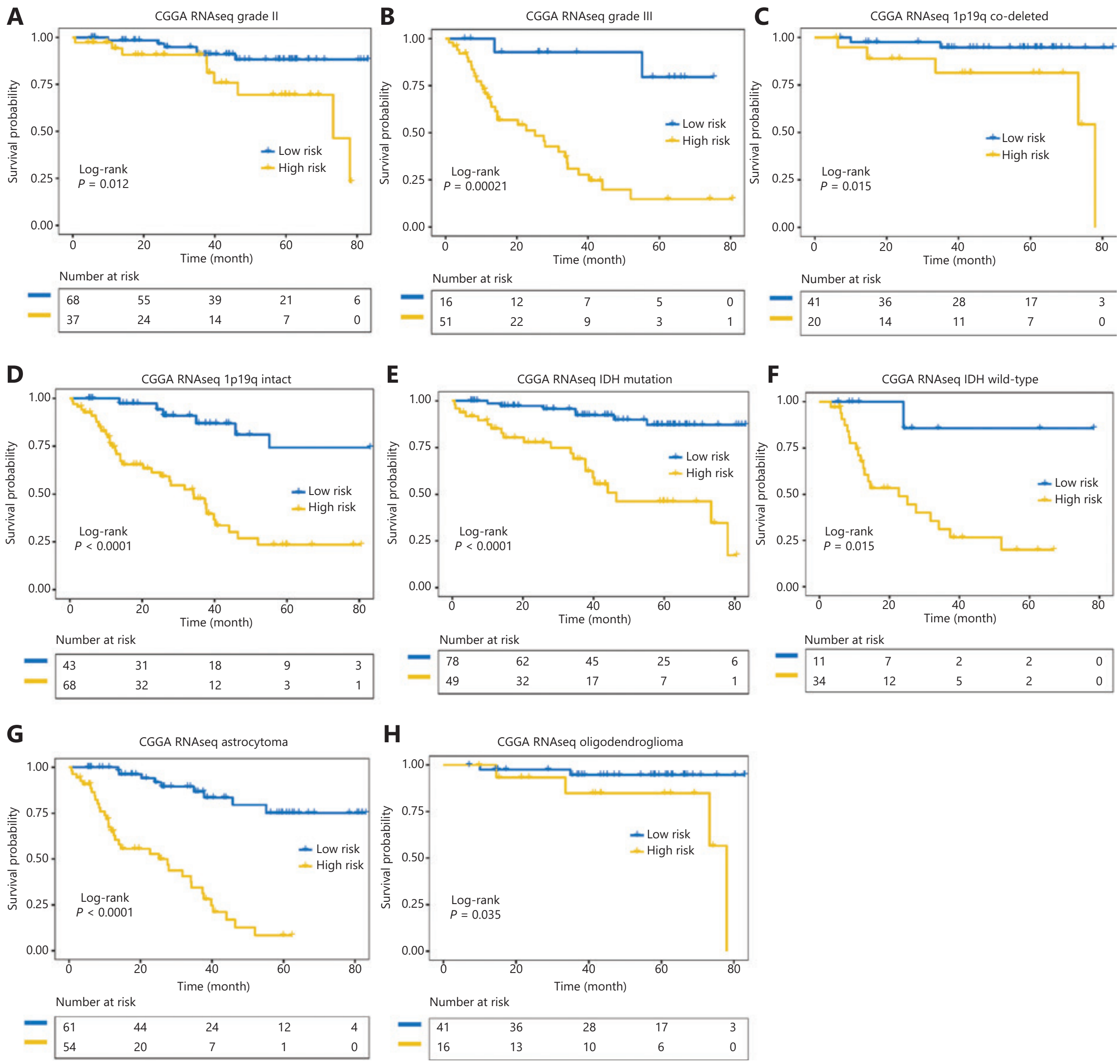

Figure S3 The clinical significance of 10 IGPs signature for LGG patients in CGGA microarray cohort. The Kaplan-Meier survival curves for LGG patients with grade II (A), grade III (B), 1p/19q codeletion (C), 1p/19q intact (D), IDH mutation (E), IDH wildtype (F), astrocytoma (G) and oligodendroglioma (H). Patients were divided into lower and higher risk score groups and *P*-value was the result of log-rank test.

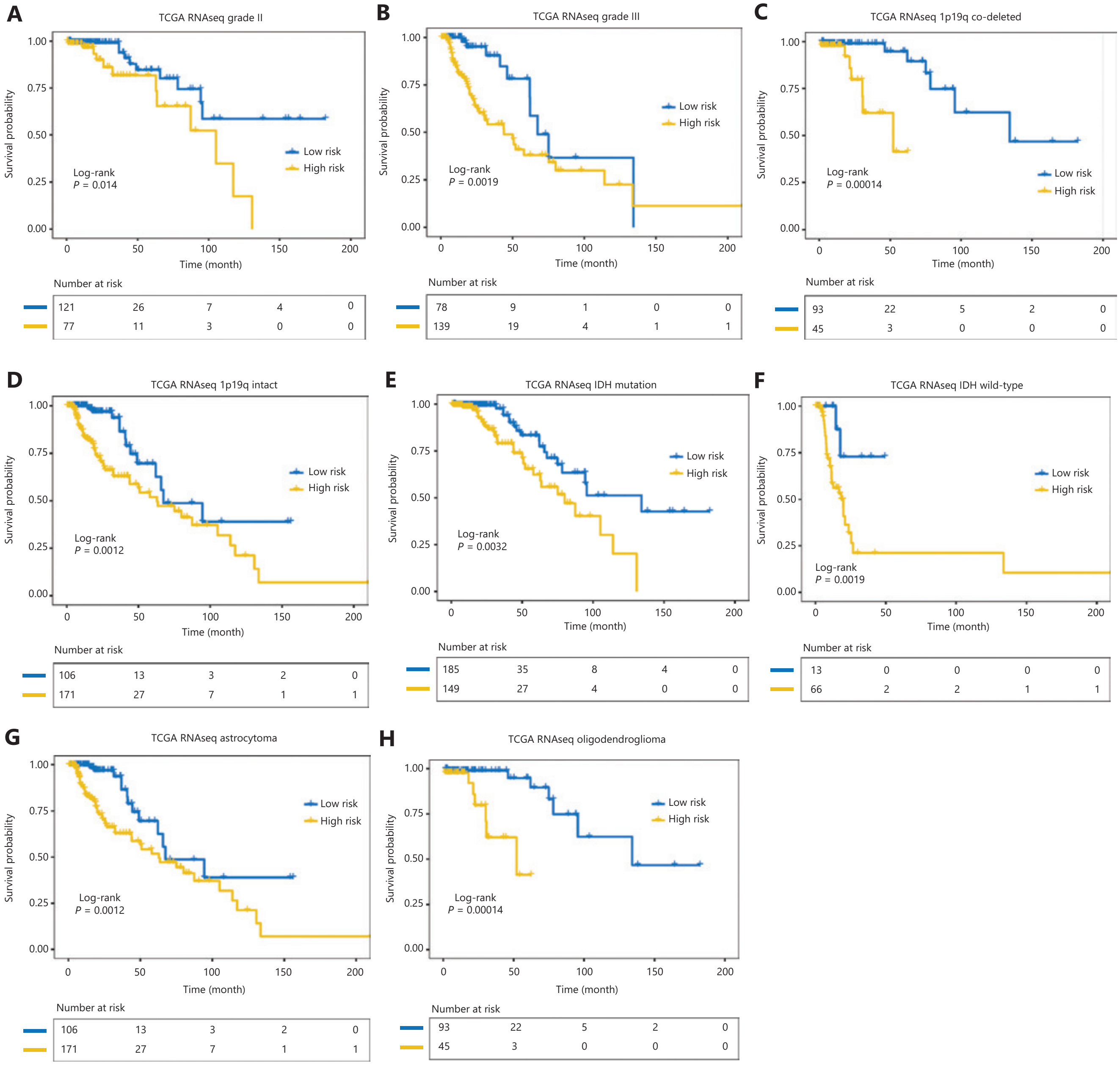

Figure S4 The clinical significance of 10 IGPs signature for LGG patients in TCGA RNAseq cohort. The Kaplan-Meier survival curves for LGG patients with grade II (A), grade III (B), 1p/19q codeletion (C), 1p/19q intact (D), IDH mutation (E), IDH wildtype (F), astrocytoma (G) and oligodendroglioma (H). Patients were divided into lower and higher risk score groups and *P*-value was the result of log-rank test..

A

CGGA RNAseq	No. of patients	*P*-value	HR (95% CI)
Total	172	1.53E–15	6.32 (4.11–9.73)
IDH status			
Mutation	127	6.71E–10	13.06 (6.16–27.67)
Wild-type	45	0.0033	3.29 (1.48–7.29)
1p19q status			
Codeleted	61	0.0002	16.18 (3.77–69.32)
Intact	111	1.91E–09	4.34 (2.74–6.88)
Grade			
Grade II	105	0.0078	5.48 (1.78–16.91)
Grade III	67	3.16E–07	4.04 (2.32–7.04)
Histology			
Astrocytoma	66	1.46E–09	3.54 (1.95–6.41)
Oligodendroglioma	39	0.0012	86.84 (1.16–65.02)
Oligoastrocytoma	67	2.72E–07	6.97 (3.34–14.54)

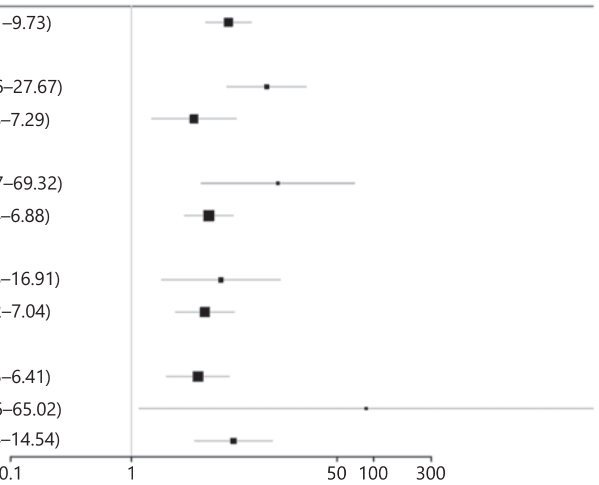

B

CGGA microarray	No. of patients	*P*-value	HR (95% CI)
Total	171	1.28E–09	7.08 (3.32 –15.09)
IDH status			
Mutation	111	1.27E–08	10.11 (4.21–24.24)
Wild-type	56	0.0009	4.23 (1.73–10.35)
1p19q status			
Codeleted	42	0.62	1.89 (0.14–24.18)
Intact	128	8.91E–10	6.35 (3.35–12.02)
Grade			
Grade II	120	0.0003	4.88 (2.01–11.83)
Grade III	51	0.0009	4.5 (1.63–12.46)
Histology			
Astrocytoma	79	8.41E–10	10.7 (4.23–27.05)
Oligodendroglioma	31	0.0281	17.99 (1.16–279.3)
Oligoastrocytoma	60	0.0003	4.39 (1.91–10.1)

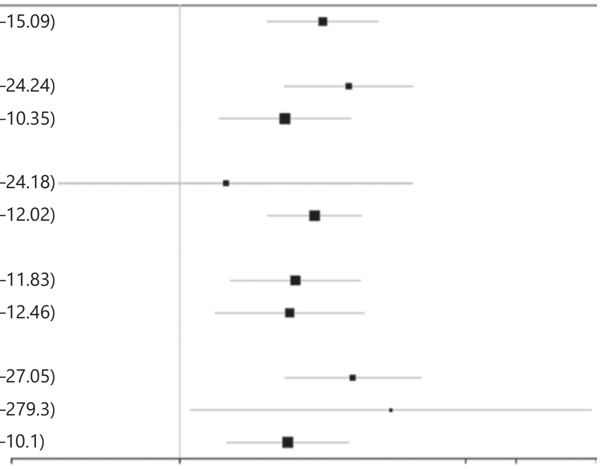

C

TCGA RNAseq	No. of patients	*P*-value	HR (95% CI)
Total	415	1.02E–06	3.57 (2.14–5.69)
IDH status			
Mutation	334	0.009	4.8 (1.47–15.64)
Wild-type	79	0.013	2.46 (1.2–5.04)
1p19q status			
Codeleted	138	0.051	12.09 (0.98–148)
Intact	277	1.39E–09	6.73 (3.63–12.49)
Grade			
Grade II	198	0.025	5.67 (1.23–26.14)
Grade III	217	3.85E–08	6.12 (3.2–11.68)
Histology			
Astrocytoma	153	1.39E–09	5.02 (2.15–11.69)
Oligodendroglioma	156	0.036	8.36 (3.36–20.81)
Oligoastrocytoma	106	0.001	41.41 (4.79–357.7)

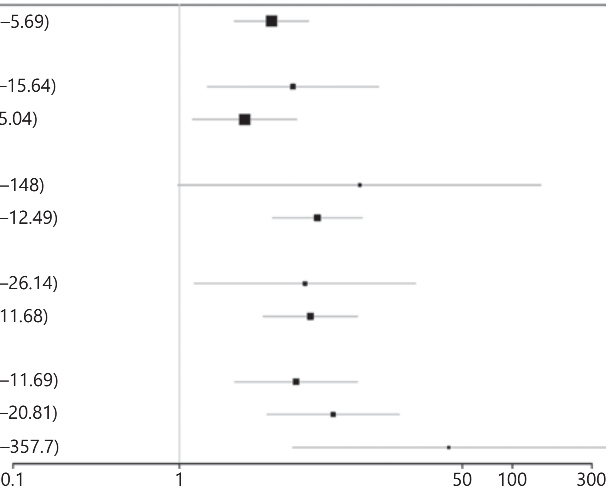

Figure S5 Univariate Cox regression analysis were applied to estimate HR values with continuous score in CGGA RNAseq cohort (A), CGGA microarray cohort (B) and TCGA RNAseq cohort (C) within subgroups for IDH status, 1p/19q status, WHO grade and histology.

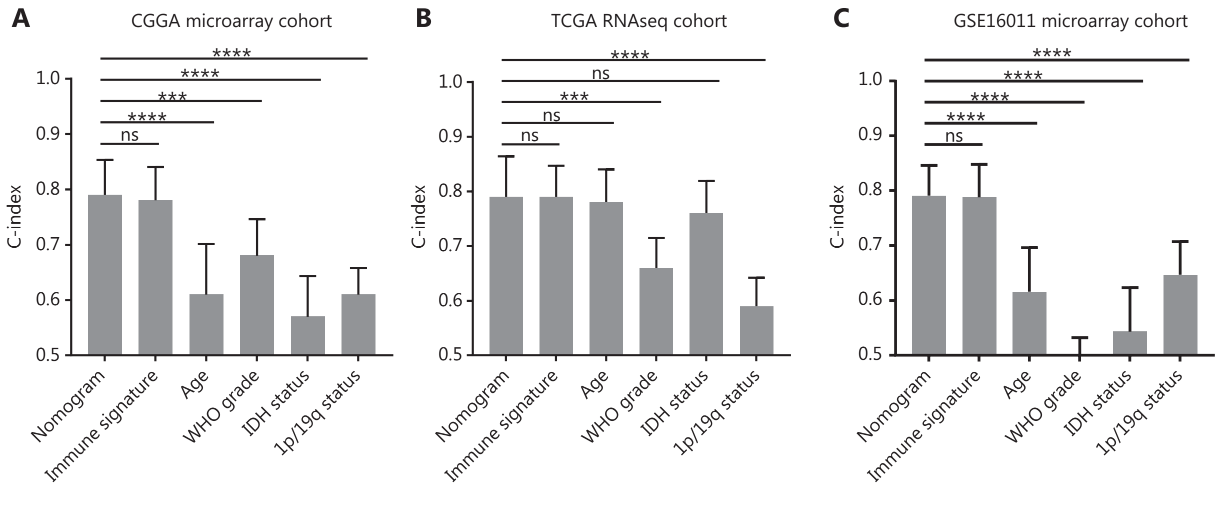

Figure S6 The c-index in predicting OS was compared between the nomogram model and other factors, including immune signature, age, WHO grade, IDH status and 1p/19q status in CGGA microarray cohort (A), TCGA RNAseq cohort (B) and GSE16011 cohort (C) (mean ± SD; ***P < 0.001, ****P < 0.0001, Student's t test).

Table S1 Prognostic factors were tested using Cox regression analysis in the 3 independent cohorts

Variable	No. of patients	CGGA RNAseq cohort				No. of patients	CGGA microarray cohort				No. of patients	TCGA RNAseq cohort			
		Univariate		Multivariate			Univariate		Multivariate			Univariate		Multivariate	
		P	HR	*P*	HR		*P*	HR	*P*	HR		*P*	HR	*P*	HR
LGG	172					171					415				
Age		<0.0001	6.3280	0.1641	1.0216		0.0017	1.0437	0.2678	1.0148		<0.0001	1.0641	<0.0001	1.0623
Gender															
Female	67	Ref				73	Ref				185	Ref			
Male	103	0.722	1.1093	0.637	1.1521	98	0.493	1.2177	0.6664	0.8730	230	0.7400	0.9274	0.7423	0.9243
Grade															
II	105	Ref				120	Ref				197	Ref			
III	67	<0.0001	6.0365	0.0007	3.4562	51	<0.0001	4.3806	0.0118	2.5652	218	<0.0001	3.2256	0.0409	1.7784
IDH status															
Wild-type	45	Ref				56	Ref				79	Ref			
Mutant	127	<0.0001	0.2516	0.0277	2.635	111	0.014	0.4953	0.7702	0.9160	334	<0.0001	0.1515	0.0049	0.4044
1p/19q status															
Intact	111	Ref				129	Ref				277	Ref			
Codel	61	<0.0001	0.1882	0.0006	0.2199	42	0.0022	0.1619	0.0328	0.2586	138	0.0015	0.3931	0.0262	0.4790
Immune signature		<0.0001	6.3285	<0.0001	4.4931		<0.0001	7.1280	0.0046	3.0747		<0.0001	8.3473	0.0213	2.300
